# PS-03 - EFFECTIVENESS OF THE MATERNAL PERTUSSIS VACCINE AGAINST PERTUSSIS INFECTION IN INFANTS IN SCOTLAND: A COHORT STUDY

**DOI:** 10.1093/pubmed/fdag046.052

**Published:** 2026-07-09

**Authors:** Dr Taimoor Hasan, Dr Ariadni Kouzeli, Mrs Aynsley Milne, Dr Daniel Chandler, Dr Sam Ghebrehewet, Dr Cheryl Gibbons

**Affiliations:** Public Health Scotland; Public Health Scotland; Public Health Scotland; Public Health Scotland; Public Health Scotland; Public Health Scotland

## Abstract

**Background:**

Pertussis can cause serious infection in infants, particularly before they are eligible for routine immunisation at eight weeks of age. To reduce this risk, a maternal pertussis vaccination programme was introduced in the UK in 2012, with vaccination recommended between 16 and 32 weeks’ gestation to provide passive protection to newborns.

**Objectives:**

We estimated the effectiveness of maternal pertussis vaccination in preventing laboratory-confirmed pertussis infection and associated hospitalisation in infants younger than eight weeks in Scotland.

**Methods:**

This was a retrospective cohort study including all singleton live births in Scotland between December 2023 and December 2024. National datasets were deterministically linked, including pregnancy and birth records, maternal vaccination data, laboratory-confirmed pertussis surveillance, and hospital admissions. The primary outcome was laboratory-confirmed pertussis infection in infants under eight weeks. Logistic regression was used to estimate odds ratios (ORs) and 95% Confidence Intervals (CIs) for infection by maternal vaccination status. A subset analysis examined the association between maternal vaccination status and pertussis-related hospitalisation among infected infants.

**Results:**

Among 48,205 singleton live births in Scotland during the study period, 33 infants aged under eight weeks had laboratory-confirmed pertussis. Twenty-five required hospital admission within 30 days of infection.

Infants with mothers vaccinated during the recommended time were significantly less likely to be infected compared to infants of unvaccinated mothers (OR: 0.13; 95% CI: 0.06-0.28). Protective effect of vaccine in infants with mothers vaccinated outside recommended time was not statistically significant (OR: 0.41; 95% CI: 0.1-1.19).

Maternal vaccination had no statistically significant effect on pertussis-associated hospitalisation (OR: 1.31; 95% CI: 0.26-7.53).

**Conclusions:**

Maternal pertussis vaccination administered within the recommended timeframe provided protection against infection in infants under eight weeks. These findings provide real-world evidence supporting timely maternal vaccination as a key strategy for protecting newborns against pertussis and for strengthening pertussis prevention in Scotland.

